# Barcoding Chrysomelidae: a resource for taxonomy and biodiversity conservation in the Mediterranean Region

**DOI:** 10.3897/zookeys.597.7241

**Published:** 2016-06-09

**Authors:** Giulia Magoga, Davide Sassi, Mauro Daccordi, Carlo Leonardi, Mostafa Mirzaei, Renato Regalin, Giuseppe Lozzia, Matteo Montagna

**Affiliations:** 1 Via Ronche di Sopra 21, 31046 Oderzo, Italy; 2 Centro di Entomologia Alpina–Università degli Studi di Milano, Via Celoria 2, 20133 Milano, Italy; 3 Museo Civico di Storia Naturale di Verona, lungadige Porta Vittoria 9, 37129 Verona, Italy; 4 Museo di Storia Naturale di Milano, Corso Venezia 55, 20121 Milano, Italy; 5 Department of Plant Protection, College of Agriculture and Natural Resources–University of Tehran, Karaj, Iran; 6 Dipartimento di Scienze per gli Alimenti, la Nutrizione e l’Ambiente–Università degli Studi di Milano, Via Celoria 2, 20133 Milano, Italy; 7 Dipartimento di Scienze Agrarie e Ambientali–Università degli Studi di Milano, Via Celoria 2, 20133 Milano, Italy

**Keywords:** Leaf beetles, molecular taxonomy, DNA barcoding, Cytochrome c oxidase subunit 1, C-bar project

## Abstract

The Mediterranean Region is one of the world’s biodiversity hot-spots, which is also characterized by high level of endemism. Approximately 2100 species of leaf beetle (Coleoptera; Chrysomelidae) are known from this area, a number that increases year after year and represents 5/6% of the known species. These features, associated with the urgent need to develop a DNA-based species identification approach for a broad spectrum of leaf beetle species, prompted us to develop a database of nucleotide sequences, with a solid taxonomic background, for all the Chrysomelidae Latreille, 1802 sensu latu inhabiting the Mediterranean region. The Mediterranean Chrysomelidae Barcoding project, which has started in 2009, involves more than fifty entomologists and molecular biologists from different European countries. Numerous collecting campaigns have been organized during the first seven years of the project, which led to the collection of more than 5000 leaf beetle specimens. In addition, during these collecting campaigns two new allochthonous species for Europe, namely *Ophraella communa* LeSage, 1986 and *Colasposoma dauricum* Mannerheim, 1849, were intercepted and some species new to science were discovered (e.g., *Pachybrachis sassii* Montagna, 2011 and *Pachybrachis holerorum* Montagna et al., 2013). DNA was extracted from 1006 specimens (~13% of the species inhabiting the Mediterranean region) and a total of 910 cox1 gene sequences were obtained (PCR amplification efficiency of 93.8%). Here we report the list of the barcoded subfamilies, genera and the number of species for which cox1 gene sequences were obtained; the metadata associated with each specimen and a list of problematic species for which marker amplification failed. In addition, the nucleotide divergence within and between species and genera was estimated and values of intraspecific nucleotide divergence greater than the average have been discussed. *Cryptocephalus quadripunctatus* G. A. Olivier, 1808, *Cryptocephalus rugicollis* G. A. Olivier, 1791 and *Exosoma lusitanicum* Linnaeus, 1767) are representatives of these cases.

## Introduction

**Figure 1.**
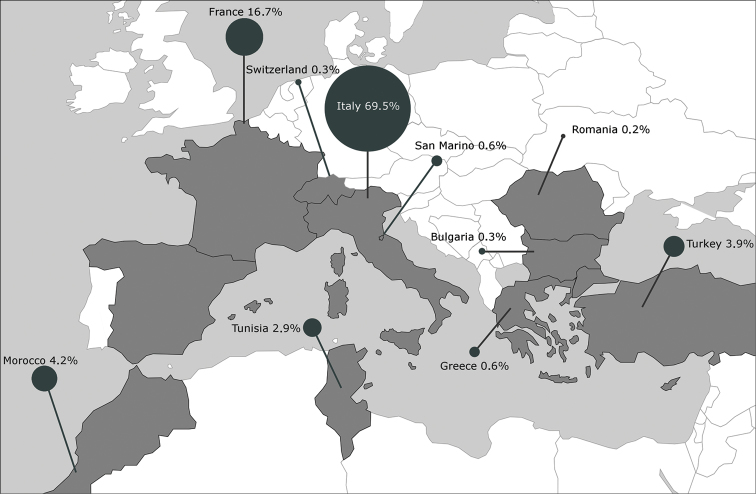
Area investigated by the Chrysomelidae Barcoding project. The countries in which were performed the collecting campaigns are reported in dark grey. The percentage of the total processed specimens is reported for each country.

In the last decades we have witnessed what has been defined as the “taxonomy impediment” (Rodman and Cody 2003) indicating the crisis in taxonomic studies due primarily to a shortage of time and taxonomists (Wheeler 2004, Wheeler et al. 2004, Wilson 2004), a situation that is made even more critical due to the decrease in the funding of natural history studies. The causes of the taxonomy crisis are many and complex, and a comprehensive analysis of this situation is beyond our purpose (see as example Boero 2001, Tautz et al. 2003). In our view, the causes can be described by the sentence …*a lack of prestige and resources that is crippling the continuing cataloguing of biodiversity* (Godfray 2002). If we consider the increased rate of species extinction (Thomas et al. 2004) amplified by climate change and habitat erosion due to exploitation by human beings the situation is worsened. A DNA-based strategy, which plays a central role in modern taxonomic studies, has been proposed by different authors as a methodology to overcome the identified problems (Tautz et al. 2002, Tautz et al. 2003, Hebert et al. 2004, Goldstein and DeSalle 2010) whilst maintaining the importance of a traditional approach mainly based on morphology. Interestingly, in a survey conducted among Coleopteran taxonomists, taxonomic initiatives based on DNA have been regarded of potential utility in solving the “taxonomy impediment”, even if a few consider it absolutely useless (Löbl 2005). Currently, in the scientific world, an agreement on the correct approach to be adopted has not yet been reached. The “gold standard” for species identification studies based on molecular markers (e.g. mitochondrial cytochrome oxidase subunit I–cox1, or the nuclear small ribosomal subunit–SSU 18S rRNA) is to develop sequence databases used as a reference, beginning with DNA extracted from type and type series specimens preserved in Museum dry collections. The main problem with this strategy is related to the conservation status of the old dry specimens; 18^th^ and 19^th^ century specimens have fragmented DNA (not easily amplified through standard PCR approaches targeting fragments of 500-700 bp) and are often infested by fungal hypha, which contaminate the insect’s genomic DNA. Even with the advent of high-throughput sequencing technologies to solve the problem of fragmented sequences, the contamination due to fungal DNA remains. Developing strategies for the acquisition and storage of molecular data to address molecular taxonomy purposes, we face another problem, which affects the DNA sequences deposited in publicly available databases, i.e. the accuracy of specimen identification. In light of these issues, an alternative strategy has been adopted in the Mediterranean Chrysomelidae Barcoding project (C-Bar). The aim of the C-Bar project is to develop a reference database of cox1 gene sequences for all the Chrysomelidae (excluding Bruchinae Latreille, 1802), the Megalopodidae Latreille, 1802 and the Orsodacnidae Thomson, 1859 (hereafter indicated as Chrysomelidae or leaf beetles sensu latu – s. l.) inhabiting the Mediterranean region. The study area of C-Bar includes all the states that possess coastline on the Mediterranean Sea or territories characterized by Mediterranean-type habitat plus Romania and Switzerland (Figure 1). Starting from the Catalogue of Palaearctic Coleoptera (Löbl and Smetana 2010), about 2100 species of Chrysomelidae s. l. (corresponding to an estimated 5/6% of all described species) are present in this area. The Mediterranean Region is one of the world’s biodiversity “hot-spots” (Myers et al. 2000, Cuttelod et al. 2008), which is characterized by exceptional concentrations of species with high levels of endemism that inhabit one of the most populated areas. The assumption of high levels of endemic species inhabiting the Mediterranean Region is also valid for leaf beetles (Biondi et al. 2013, Sassi 2006). Although the Mediterranean region has been the subject of investigation by generations of entomologists, knowledge of Chrysomelidae inhabiting this area is far from being fully known. The number of leaf beetle species new to science described from the Mediterranean region in the last decades, associated with the fact that they are widespread among different genera, confirms the need to increase the effort in biodiversity-based studies (e.g. *Cryptocephalus* O.F. Muller, 1764, *Chrysolina* Motschulsky, 1860, *Gonioctena* Motschulsky, 1860, *Longitarsus* Berthold, 1827, *Psylliodes* Berthold, 1827, *Colaspidea* Laporte de Castelnau, 1833; Bastazo 1997, Biondi 1997, Sassi 2001, Leonardi 2007, Daccordi and Ruffo 2005, Baviera 2007, Vela and Bastazo 2012, Zoia 2014).

In this project are involved taxonomists, specialized in different leaf beetle clades, in order to guarantee the accurate specimen identification. In our view, the adoption of this strategy is a way to bring together traditional (intended as based on morphology) and molecular taxonomy in order to tentatively overcome the “taxonomy impediment” (Rodman and Cody 2003).

The purpose of this paper is to report the preliminary results achieved during the first seven years of the project in order to show the potential of a cooperation between molecular biologists and traditional taxonomists. In particular, we report: *i*) the method adopted and issues arisen in the development of the sequence dataset; *ii*) the list of subfamilies, genera and the number of species for which cox1 gene sequences were obtained; *iii*) the metadata associated with the processed organisms; *iv*) mean values of intraspecific and interspecific nucleotide divergence *v*) the new species described and the important faunistic findings.

## Materials and methods

### Specimen collection and identification

More than 50 entomologists, from different European Countries, have joined the C-Bar project and have actively participated in samples collection. During the first seven years of the project (from 2009 to 2015) numerous collecting campaigns were organized from March to September of each year. The specimens were collected using different methods: from the vegetation by sweep net or by beating sheet, and directly by hand in specific habitats (e.g. under stones or digging the host plant roots). All the collected specimens were placed in 5 ml vials filled with absolute ethanol in order to preserve the genomic DNA. Within an hour of specimen collection, the mixture in the vials was replaced with fresh absolute ethanol in order to obtain better sample dehydration and preservation for long-term storage. Each vial was preserved at -20°C and was labeled by a unique identifier plus other metadata related to the sampling locality (i.e. Country, Province, Region, exact site, latitude, longitude and elevation), the date of collection, the collector/s and other ecological information related to the specimens.

Specimen manipulation and dissection (when necessary) were completed with the auxiliary use of a stereomicroscope. Images of the specimen habitus were acquired by a reflex camera (Canon EOS 450D, macro objective 60 mm or 100 mm with a set of macro extension tubes) or with Axiocam 506 mounted on Zeiss Axio Zoom V16. The specimens were morphologically identified by Italian taxonomists expert in different leaf beetle clades (most of them are listed among the authors of the present article). The nomenclature adopted in the C-bar project follows the work of Bouchard et al. (2011) at the levels of family and subfamily, while at the levels of genus and species was adopted the recently published Catalogue of Palaearctic Coleoptera–Chrysomeloidea (Löbl and Smetana 2010).

### DNA extraction, PCRs and sequence quality control

DNA extraction was performed in two different ways since it took place in different laboratories (Biodiversity Institute of Ontario, University of Guelph and Department of Agricultural and Environmental Sciences, Università degli Studi di Milano): for 950 samples the DNA was extracted from one hind leg while for the 56 remaining samples the DNA was extracted from the whole specimen, after the removal of the abdomen. The latter procedure ensures to keep specimen morphology intact. In both cases, DNA was purified using the Qiagen DNeasy Blood and Tissue Kit (Qiagen, Hilden, Germany). Here we describe the adopted non-destructive procedure: the specimen was taken off from absolute ethanol and dried in single 1.5 ml vials for 45 minutes at 30°C; after the removal of the abdomen with the use of sterile pins and tweezers the specimen was placed in 180 µL of ATL lysis buffer (Qiagen) with 200 ng/mL proteinase K (Sigma Aldrich, St. Louis, MO, USA) at 56°C for 12 hours. The following steps of the DNA extraction were performed according to the manufacturer’s instructions of Qiagen DNeasy Blood and Tissue Kit. After DNA extraction, the specimens were dry mounted on pins together with genitalia and kept for future reference. A quote of the extracted DNA was preserved in the C-bar DNA library at -80°C for long term storage and a rate was preserved at -20° in order to perform the following amplifications. A fragment of 658 bp at the 5’-end of the mitochondrial cytochrome c oxidase subunit 1 gene (cox1) was amplified with primers LCO1490 5’-GGT CAA CAA ATC ATA AAG ATA TTG G / HCO2198 5’-TAA ACT TCA GGG TGA CCA AAA AAT CA (Folmer et al. 1994). When this pair of primers resulted in unsuccessful amplification of the target marker, other primers amplifying the same gene region were used, i.e. LepF1 5’-ATT CAA CCA ATC ATA AAG ATA TTG G / LepR1 5’-TAA ACT TCT GGA TGT CCA AAA AAT CA (Hebert et al. 2004). Successful amplifications were determined by gel electrophoresis. PCR products were directly sequenced on both strands using the marker-specific primers from ABI technology (Applied Biosystems, Foster City, CA, USA). The obtained sequences were edited using Geneious R8 (Biomatters Ltd., Auckland, New Zealand) and primers, pseudogenes and contaminations removed. Finally, they were deposited in the Bold Systems (Ratnasingham and Hebert 2007) and in the European Nucleotide Archive (Montagna et al. under revision).

### Intraspecific and intrageneric nucleotide divergence

The obtained cox1 gene sequences were aligned at codon level using MUSCLE (Edgar 2004) with default parameters. A pairwise nucleotide distance matrix was estimated starting from the aligned sequences implementing the Kimura-two-parameter (K2P) model (Kimura 1980), considered as an adequate evolutionary nucleotide model when p-distances between sequences are low (Nei and Kumar 2000). The nucleotide distance matrix was used for the calculation of the mean intraspecific and interspecific nucleotide distances and for the calculation of mean intrageneric distance; these analyses were performed using the R package Spider (Brown et al. 2012). We also calculated nucleotide intraspecific distances for some species with a wide range of distribution.

## Results and discussion

Until now, C-Bar collecting campaigns have investigated some areas of Bulgaria, France, Greece, Italy, Morocco, Romania, Spain, Switzerland, Turkey and Tunisia (Figure 1). The sampling efforts that have been accomplished until now led to the collection of more than 5000 Chrysomelidae specimens. During the identification process, some specimens of previously unknown species were recognized, these samples were used for the description of the following species: *Pachybrachis sassii* (Montagna 2011) from the Giglio Island in the Tuscan Archipelago; *Pachybrachis holerorum* (Montagna et al. 2013) from the Northern Apennines and *Oulema mauroi* Bezděk & Baselga, 2015, from Northen Italy. Other samples collected during the C-Bar collecting campaigns were used in a revision of Colaspidea genus that led to the description of seven new species (Zoia 2014). All these new taxa were formally described by a traditional morphological approach, in some cases molecular data were added to confirm the existence of the new species. Besides the discovery of new taxa, two allochthonous species new to Europe, namely *Ophraella communa* (Boriani et al. 2013) and *Colasposoma dauricum* (Montagna et al. in press), were intercepted. *Ophraella communa* is a leaf beetle of Nearctic origin accidentally introduced in 1996 in Taiwan (Wang and Chiang 1998) and Japan (Takizawa et al. 1999); the species rapidly spread in East Asia and few years ago we intercepted it in the Northern part of Italy (Boriani et al. 2013). *Colasposoma dauricum* is a species originally present in the North and Central-East of Asia, it has never been observed out of its original range until our interception in 2011 in Piedmont (North of Italy).

Among the collected samples, the DNA was extracted from 1006 specimens and PCRs targeting a fragment of the cox1 gene performed. PCRs with the selected primer pairs lead to successful amplification in 93.8% of the cases (62 specimens failed the amplification). Among the specimens for which the amplification failed, 43 specimens belong to the subfamily Cryptocephalinae Gyllenhaal, 1813: 18 species of *Cryptocephalus* (40 specimens); interestingly cox1 sequences have never been obtained for *Cryptocephalus therondi* Franz, 1949, *Cryptocephalus cantabricus* Franz, 1958 and *Cryptocephalus etruscus* Sassi, 1995. We can hypothesize the presence of mutations in the annealing region of the used primers. Sequences obtained from *Clytra laeviuscula* Ratzeburg, 1837, *Clytra quadripunctata* Linnaeus, 1758, *Cryptocephalus cristula* Dufour, 1843, *Cryptocephalus octoguttatus* Linnaeus, 1767, *Lachnaia tristigma* Lacordaire, 1848 and *Oomorphus concolor* Sturm, 1807 did not possess an open reading frame and were thus considered as nuclear pseudogenes. Twenty-seven sequences were discarded because of contamination from exogenous DNA. A total of 910 cox1 sequences (267 species corresponding to ~13% of those inhabiting the Mediterranean region) were obtained, the size of the sequences was > 400 bp in ~99% of the cases.

We observed that only two species, namely *Cryptocephalus violaceus* Laicharting, 1781 and *Cryptocephalus duplicatus* Suffrian, 1845, sharing the same haplotype can not be discriminated through DNA barcoding. In this and in similar cases a barcoding failure can be confirmed only ensuring the correct identification of the samples by expert taxonomists. Therefore 99.3% of the species (265) for which we obtained cox1 sequences possessed unique haplotypes, allowing their molecular identification. The mean intraspecific nucleotide distance value is of 2%, while the mean interspecific and intrageneric distances result of, respectively, 25.2% and of 19.8%. The obtained intraspecific value are higher than that inferred in a previous study on Coleoptera (Pentinsaari et al. 2014). This results might be the effect of geographical distances among localities of collection of co-specific specimens; a possible alternative explanation is the presence of cryptic species. Among the species showing high intraspecific nucleotide distance noteworthy are the cases of *Cryptocephalus rugicollis* (2.8% [0%, 5.5%]), *Exosoma lusitanicum* (6.7% [0.2%, 9.2%]) and *Cryptocephalus quadripunctatus* that shows a mean intraspecific distance (3% [0%, 4.9%]). To test the formulated hypotheses further analyses, including the use of other mitochondrial and nuclear markers as well as a wider sample of specimens, are required.

Among the nine subfamilies for which cox1 sequences were obtained (Table 1), Cryptocephalinae and Galerucinae Latreille, 1802 were better represented. In the first subfamily are listed 111 species (83 species of Cryptocephalini Gyllenhaal, 1813 and 28 of Clytrini Lacordaire, 1848, 426 specimens in total) while the second counts 88 species (24 species of Galerucini Latreille, 1802 and 64 of Alticini Spinola, 1844, 274 specimens in total). The unbalanced sampling towards Cryptocephalini, which in some way might affect the obtained results, could be explained by the fact that most of the C-bar specimens have been collected by Sassi and Montagna, which mainly work on this clade and are likely to have developed collecting strategies that increase their sampling (Figure 1).

**Table 1.** List of the barcoded subfamilies and genera with the number of species and specimens belonging to each taxon.

Subfamily	Genus	*N_s_^a^*	*^b^N_spec_*	*N^b^*
Zeugophorinae Böving & Craighead, 1931	*Zeugophora* Kunze, 1818	1	1	1
Orsodacninae Thomson, 1859	*Orsodacne* Latreille, 1802	3	7	2.3
Donacinae Kirby, 1837	*Donacia* Fabricius, 1775	2	6	3
Criocerinae Latreille, 1804	*Crioceris* Muller, 1764	3	18	3
	*Lilioceris* Reitter, 1912	1		
	*Lema* Fabricius, 1798	1		
	*Oulema* Gozis, 1886	1		
Cassidinae Gyllenhal, 1813	*Cassida* Linnaeus, 1758	14	61	3.4
	*Hypocassida* Weise, 1893	2		
	*Hispa* Linnaeus, 1767	1		
	*Dicladispa* Gestro, 1897	1		
Chrysomelinae Latreille, 1802	*Chrysolina* Motschulsky, 1860	13	117	3.4
	*Chrysomela* Linnaeus, 1758	3		
	*Entomoscelis* Chevrolat, 1836	1		
	*Gastrophysa* Chevrolat, 1836	1		
	*Gonioctena* Motschulsky, 1860	3		
	*Oreina* Chevrolat, 1836	6		
	*Plagiosterna* Motschulsky, 1860	1		
	*Phratora* Chevrolat, 1836	1		
	*Plagiodera* Chevrolat, 1836	1		
	*Prasocuris* Latreille, 1802	1		
	*Timarcha* Latreille, 1829	3		
Galerucinae Latreille, 1802	*Agelastica* Chevrolat, 1836	1	274	3.1
	*Arima* Chapuis, 1875	1		
	*Calomicrus* Stephens, 1831	3		
	*Exosoma* Jacoby, 1903	2		
	*Diabrotica* Chevrolat, 1836	1		
	*Galeruca* Geoffroy, 1762	5		
	*Galerucella* Crotch, 1873	3		
	*Lochmaea* Weise, 1883	2		
	*Luperus* Geoffroy, 1762	6		
	*Nymphius* Weise, 1900	2		
	*Sermylassa* Reitter, 1913	1		
	*Altica* Muller, 1764	4		
	*Aphthona* Chevrolat, 1842	6		
	*Argopus* Fischer von Waldheim, 1824	1		
	*Arrhenocoela* Foudras, 1860	1		
	*Chaetocnema* Stephens, 1831	2		
	*Crepidodera* Chevrolat, 1836	5		
	*Derocrepis* Weise, 1886	2		
	*Dibolia* Latreille, 1829	2		
	*Epitrix* Foudras, 1860	1		
	*Hermaeophaga* Foudras, 1860	1		
	*Hippuriphila* Foudras, 1860	1		
	*Longitarsus* Berthold, 1827	9		
	*Lythraria* Bedel, 1897	1		
	*Neocrepidodera* Heikertinger, 1911	6		
	*Phyllotreta* Chevrolat, 1836	4		
	*Podagrica* Chevrolat, 1836	1		
	*Psylliodes* Berthold, 1827	12		
	*Sphaeroderma* Stephens, 1831	2		
Cryptocephalinae Gyllenhal, 1813	*Cryptocephalus* Geoffroy, 1762	73	426	3.8
	*Pachybarchis* Chevrolat, 1836	8		
	*Stylosomus* Suffrian, 1848	2		
	*Clytra* Laicharting, 1781	4		
	*Coptocephala* Chevrolat, 1836	3		
	*Labidostomis* Chevrolat, 1836	10		
	*Lachnaia* Chevrolat, 1836	3		
	*Macrolenes* Chevrolat, 1836	1		
	*Smaragdina* Chevrolat, 1836	7		
	*Tituboea* Lacordaire, 1848	1		
Eumolpinae Hope, 1840	*Chrysochus* Chevrolat, 1836	1	5	1.7
	*Colaspidea* Laporte de Castelnau, 1833	1		
	*Macrocoma* Chapuis, 1874	1		

a *N_s_* indicates the number of barcoded species

b *N_spec_* and *N* indicates respectively the total number and the average number of barcoded specimens belonging to each subfamily

The metadata related to the specimens (i.e., specimen identification, collection identifier, collecting date, state, province, exact site of collection, latitude, longitude, elevation and collector/s) from which cox1 gene sequences were obtained, are available in a web site dedicated to the project (http://www.c-bar.org). Regarding the specimens collected within Italian administrative boundaries the metadata associated with the specimens are also available in the Biodiversity Database and GIS platform of the Italian National Network of Biodiversity. These faunistic data are useful because increase the awareness of species presence and distribution in the sampled area.

## Conclusion

In this paper, we report that C-Bar project, besides having produced useful data for molecular taxonomy (cox1 sequences were obtained for about 13% of the species inhabiting the investigated area), has obtained important results also from the viewpoint of the classical taxonomy leading to the morphological description of same new species of Chrysomelidae. A further important achievement has been the interception of allochthonous species. These results have been obtained only thanks to the cooperation amongst the taxonomists specialized in different leaf beetle clades, which have ensured the correct identification of samples, the people involved in the extensive collecting campaigns and the molecular biologists.

The promising preliminary results that have been obtained encourage us to continue with this project since they strongly confirm the urgent need to increase the efforts in faunistic studies to uncover the real biodiversity of leaf beetles inhabiting the Mediterranean region. For these reasons, we are confident that the aim of C-bar project of developing a repository of cox1 sequences for the majority of the species of Chrysomelidae s. l. inhabiting the Mediterranean region may be achieved in the near future.

In conclusion, as demonstrated by the relevant results obtained during the first years of the project, we believe that DNA barcoding projects, when developed with the participation of taxonomists and molecular biologists, represent an opportunity to bring together two different worlds and may be considered the driving force able to revive interest in what can be regarded as the milestone of biological studies that is a-taxonomy, helping to fill the “taxonomy impediment”.
